# Micellar Iron Oxide Nanoparticles Coated with Anti-Tumor Glycosides

**DOI:** 10.3390/nano8080567

**Published:** 2018-07-25

**Authors:** Hugo Groult, Isabel García-Álvarez, Lorenzo Romero-Ramírez, Manuel Nieto-Sampedro, Fernando Herranz, Alfonso Fernández-Mayoralas, Jesús Ruiz-Cabello

**Affiliations:** 1 UMR CNRS 7266 LIENSs, Approches Moléculaires Environnement-Santé Environnement (AMES), Avenue Michel Crepeau, Université de La Rochelle, 86073 La Rochelle, France; hugo.groult@univ-lr.fr; 2 Department of Biological Engineering, Massachussetts Institute of Technology, 77 Massachusetts Avenue, Cambridge, MA 02139, USA; ialvarez@mit.edu; 3 Faculty of Experimental Sciences, Francisco de Vitoria University (UFV), Ctra. Pozuelo-Majadahonda Km 1.800, Pozuelo de Alarcón, 28223 Madrid, Spain; 4 Hospital Nacional de Parapléjicos, SESCAM, Finca la Peraleda s/n, 45004 Toledo, Spain; lromeroramirez@sescam.jccm.es (L.R.-R.); mnietosampedro@gmail.com (M.N.-S.); 5 Instituto Cajal, CSIC, Avda. Doctor Arce 37, 28002 Madrid, Spain; 6 Advanced Imaging Unit, Centro Nacional de Investigaciones Cardiovasculares Carlos III (CNIC), 28029 Madrid, Spain; fernando.herranz@cnic.es; 7 Instituto de Química Orgánica General, CSIC, Juan de la Cierva, 3, 28006 Madrid, Spain; alfonso.mayoralas@csic.es; 8 CIC biomaGUNE and Ciber de Enfermedades Respiratorias, Parque Científico y Tecnológico de Gipuzkoa, Paseo Miramón 182, 20014 Donostia/San Sebastián, Gipuzkoa, Spain; 9 CIBER de Enfermedades Respiratorias (CIBERES), 28029 Madrid, Spain

**Keywords:** glycoside, iron oxide nanoparticles, nanomicelles, antitumoral

## Abstract

The synthesis procedure of nanoparticles based on thermal degradation produces organic solvent dispersible iron oxide nanoparticles (OA-IONP) with oleic acid coating and unique physicochemical properties of the core. Some glycosides with hydrophilic sugar moieties bound to oleyl hydrophobic chains have antimitotic activity on cancer cells but reduced in vivo applications because of the intrinsic low solubility in physiological media, and are prone to enzymatic hydrolysis. In this manuscript, we have synthetized and characterized OA-IONP-based micelles encapsulated within amphiphilic bioactive glycosides. The glycoside-coated IONP micelles were tested as Magnetic Resonance Imaging (MRI) contrast agents as well as antimitotics on rat glioma (C6) and human lung carcinoma (A549) cell lines. Micelle antimitotic activity was compared with the activity of the corresponding free glycosides. In general, all OA-IONP-based micellar formulations of these glycosides maintained their anti-tumor effects, and, in one case, showed an unusual therapeutic improvement. Finally, the micelles presented optimal relaxometric properties for their use as T2-weighed MRI contrast agents. Our results suggest that these bioactive hydrophilic nano-formulations are theranostic agents with synergistic properties obtained from two entities, which separately are not ready for in vivo applications, and strengthen the possibility of using biomolecules as both a coating for OA-IONP micellar stabilization and as drugs for therapy.

## 1. Introduction

Nanotechnology has important applications in biomedicine, mainly in the field of cancer treatment [1]. The singular structural properties of nanoparticles (NP) permit the design of drug delivery platforms that easily answer the main problems of chemotherapeutically based tumor treatments, especially adding the specificity of an anti-tumoral drug without disturbing healthy cells and tissues [2,3]. The use of nanotechnology introduces targeting options either by passive accumulation of the NP inside tumor cells resulting from enhanced permeability and retention effect (EPR) or active ligand-mediated targeting [4,5,6]. Suitable targeting agents often improve pharmacokinetics, e.g., local delivery with improved dosing, membrane permeation as well as a reduced toxicity or minimum side effects by specific release of the therapeutic cargo in the target tissues [7,8]. Within the large number of nanomaterials, superparamagnetic iron oxides (IONP) are particularly interesting for their capability to direct drugs at the pathological area mediated by an external, internally implanted, or even spatially directed magnetic field [9,10]. Furthermore, IONP can act as contrast agents for MRI, providing diagnostic capabilities to the therapeutic agent [11,12]. Several studies of magnetically driven drug targeting in small animal models and clinical trials have successfully reported partial remission of the tumor [13,14]. Finally, there is a growing interest in the use of IONP for hyperthermia applications [15].

A family of synthetic glycosides was proposed as inhibitors of glioma and adenocarcinoma cell proliferation [16,17]. These glycosides are derivatives of *N*-acyl-d-glucosamine and previous results indicate that their activity increase when a long hydrocarbon chain was present at the C-1 position of the glycoside moiety. Other groups have shown that the conjugation of a sugar with an oleyl chain improves the antitumoral activity of the conjugate [18]. The proposed mechanism of growth inhibition suggests alterations in lipid metabolism. Indeed, it has been shown that glycoside-treatments caused important changes in the level of glycosphingolipids [19,20], which have regulatory roles in tumor progression and are involved in pathways of cell death or proliferation [21,22,23]. However, preliminary in vivo experiments with the best candidates offered modest results [24]. Because of their long alkyl chain, the compounds were poorly soluble in aqueous physiological media and some of them were also subjected to enzymatic degradation, which reduced their biological stability [25]. Consequently, it was only possible to use intratumoral administration of drugs dispersed in a dimethyl sulfoxide (DMSO)/H_2_O mixture with Bovine Serum Albumin (BSA) as carrier. Only one enzyme-resistant thioglycoside derivative showed significant tumor growth inhibitory activity with high repetitive doses [24,26]. These considerations indicate that IONP-based drug delivery systems may be effective for both stabilizing and targeting of the bioactive glycosides to the tumor site, leading to improved therapeutic activity.

The therapeutic agents in most IONP drug delivery systems are either covalently bound or electrostatically adsorbed onto the IONP surface, dispersed into a polymer matrix, or encapsulated into amphiphilic nanostructures [5,27]. Commonly, all these solutions require a pre-existing hydrophilic coating with functional moieties on the IONP scaffold. For the sake of simplicity, the method based on co-precipitation that yields aqueous IONP is still preferred due to the possibility of further surface tailoring, although the NP obtained by this method are of low quality [28]. Moreover, additional steps are then required for the integration of the drugs [29]. The thermal degradation method provides the best solution in terms of physicochemical properties (size, size dispersion, crystallinity, reproducibility) of the iron oxide cores but yields IONP only stable in organic solvent. Consequently an additional step is required for aqueous stabilization of the NP before any integration of drugs [30]. Here, we address the possibility of directly stabilizing oleic acid-coated iron oxide nanoparticles (OA-IONP) prepared by thermal degradation through micellation with the bioactive glycosides described before. In these colloidal structures, the hydrophobic alkyl glycoside tails surround the aliphatic chain of the OA-coated IONP through hydrophobic interactions, while the hydrophilic sugar is distributed on the outer surface of the micelle, thus improving water-dispersibility by covering the hydrophobic layer. This configuration where the therapeutic cargo also functions as the micellar coating requires additional tests to study the viability of the drug delivery system. As a first step, it must be tested whether the bioactive coatings fulfill the requirements of the resulting hydrophilic matrix for the in vivo use of the probes (i.e., prevented opsonization, improved colloidal stability, biocompatibility, and suitable relaxometric properties for imaging applications). Then it is key to monitor whether the anti-tumor activity is preserved in the micellar formulations of these therapeutic compounds.

## 2. Materials and Methods

### 2.1. Materials and General Procedures

All chemicals for the preparation of the glycosides were of reagent grade or higher and were purchased from commercial suppliers or purified by standard techniques as described in the previously published methods. Thin-layer chromatography (TLC) was performed on aluminum sheets 60 F254 Merck silica gel, and compounds were visualized by ultraviolet (UV) irradiation or by treatment with Ce_2_MoO_4_ or 5% H_2_SO_4_ solutions in ethanol, followed by heating. Flash column chromatography was performed using thick-walled columns filled with silica gel (Merck 60: 0.040–0.063 mm). In all cases, the eluent used is indicated, and the solvent ratios refer to volume. Mass spectrometry was performed with an Agilent 6500 Accurate Mass Q-TOF spectrometer (Santa Clara, CA, USA) with an electrospray source. The purity of all compounds was >95% as determined by elemental analyses using a Heraus CHN-O analyzer (Cologne, Germany). All the chemicals for the preparation of the oleic acid-coated iron oxide nanoparticles OA-IONP were purchased from Sigma-Aldrich Co. (St. Louis, MO, USA).

### 2.2. Synthesis

#### 2.2.1. Synthesis of the Oleic Acid-Coated Iron Oxide Nanoparticles, OA-IONP

The preparation of OA-IONP was based on a thermal degradation method we published before [7]. Briefly, to a three-neck flask was added a mixture of iron acetylacetonate (0.71 g, 2 mmol), 1,2-hexadecanediol (2.38 g, 10 mmol), oleic acid (1.69 g, 6 mmol), oleylamine (1.60 g, 6 mmol) and phenyl ether (20 mL). The reaction was kept under inert atmosphere (N_2_) and heated up to 200 °C with mechanical stirring. Magnetic stirring was avoided to not interfere with the magnetic properties of the iron oxide nanoparticles. Once the desired temperature stage was reached, it was kept for 120 min. Subsequently the mixture was brought to reflux during 30 min at 254 °C before being cooled to room temperature (RT). After that, ethanol (EtOH) was added and the resulting solution was centrifuged at 8500 rpm. Obtained supernatant was next discarded to remove side products. This purification step was repeated three times, and finally OA-IONP were re-dispersed in n-hexane (20 mL) and oleic acid (0.05 mL). This suspension was centrifuged at 8500 rpm to remove aggregates and obtain the final OA-IONP colloid.

#### 2.2.2. Synthesis of GC22, IG20 and TFA-GC22

The glycosides GC22, IG20, TFA-GC22 were synthesized according to previous reported procedures [16,17,31]. Briefly for GC22 (Oleyl 2-Acetamido-2-deoxy-α-d-glucopyraroside), *N*-Acetyl-d-glucosamine (4.32 g, 19.5 mmol) was dissolved in oleic alcohol (85%) (28 mL, 76 mmol) under argon and treated with the acid catalyst H_2_SO_4_ adsorbed on silica (440 mg). The reaction mixture was stirred at 180 °C for 1 h. After this time, the mixture was cooled at room temperature and diluted with methanol. Then, the catalyst filtrated and compound GC22 was obtained by silica gel column chromatography Ethyl acetate-Methanol (AcOEt-MeOH) 10:0 to 10:1 as a solid (3.3 g, 36%). Two further crystallizations afforded GC22 with higher purity (1.33 g, 14%). For IG20 (oleyl 2-acetamido-2-deoxy-6-*O*-(oxosulfonyl)-α-d-glucopyranoside potassium salt), glycoside GC22 (521 mg, 1.1 mmol) was dried under vacuum, dissolved in anhydrous pyridine (1.4 mL) and cooled to 0 °C. Then, SO_3_-pyridine complex (210 mg, 1.32 mmol) was added and the reaction mixture was stirred under argon at 0 °C for 90 min. Then it was stirred at room temperature for 2 h. After this time, the solvent was removed on vacuum and the residue was dissolved in MeOH-H_2_O (2:1, 25 mL), neutralized with a 0.5 M KOH solution and concentrated in vacuum. The residue was purified by silica gel column chromatography (CH_2_Cl_2_/MeOH, 4:1 to 2:1) to give IG20 as a white solid (525 mg, 81%). For TFA-GC22 (oleyl 2-deoxy-2-trifluoroacetamido-α-d-glucopyranoside), *N*-trifluoroacetyl-d-glucosamine (1.18 g, 4.29 mmol), prepared as described in the literature [32], was dissolved in 85% (*v*/*v*) oleic alcohol (mL, 21.4 mmol) and treated with 35% (*w*/*w*) H_2_SO_4_-silica (236 mg). This mixture was stirred at 180 °C under argon for 20 min (TLC: ethyl acetate). After this period, the mixture was cooled at 25 °C and purified by silica gel column chromatography (hexane-ethyl acetate, 2:1 to 0:1) to obtain 29% yield TFA-GC22 (604 mg) as a white solid.

#### 2.2.3. Synthesis of Glycosides-Coated IONP Micelles GC22-IONP, IG20-IONP, TFA-GC22-IONP

The glycosides GC22, IG20, TFA-GC22 (10 to 20 mg dissolved in 1 mL of ethanol was initially dispersed in 15 mL of 5 mM phosphate buffer at pH = 7.2 One milliliter of the OA-IONP suspension in hexane (with 10–15 mg·mL^−1^ iron concentration) was then added to the solution and the resultant mixture was sonicated for 20 min at 37 °C under robust stirring (Branson 250 digital Ultranofier, 42 ± 6 KHz, Danbury, CT, USA). The resultant oil in water (o/w) emulsion was for an additional 1 h sonication to remove all traces of hexane and ethanol resulting in a homogenous micellar aqueous suspension. Aggregates were filtrated (0.22 μm, polyvinylidene difluoride membrane) and the excess of free glycoside eliminated by gel filtration in a PD-10 column (GE Healthcare, Milwaukee, WI, USA).

### 2.3. Physicochemical Characterization of the Oleic Acid-Coated IONP (OA-IONP) and the Glycosides-Coated IONP Micelles GC22-IONP, IG20-IONP, TFA-GC22-IONP

Zetasizer Nano ZS90 (Malvern Instruments, Malvern, UK) was used to measure the hydrodynamic size, polydispersity index and zeta potential of the IONP micelles. JEOL-3000 (FXII, 300-keV, Akishima, Tokio, Japan) was used to determine the shape and size of the IONP cores. Samples were prepared as follows, a drop of glycoside-coated IONP micelles diluted solution was put on a carbon-coated grid and dried at RT for one day. Spectrum 400 Series spectrometer (Perkin Elmer, Waltham, MA, USA) was used to obtain the Fourier transformed infrared spectroscopy (FTIR) spectra from the average of 32 interferograms at a 1 cm^−1^ resolution. TG/ATD 320 U, SSC 5200 (Seiko Instruments, Minato, Tokio, Japan) was used for thermogravimetric analysis (TGA) The dried IONP micelles were heated from RT to 1000 °C at a rate of 10 °C per min and under an airflow of 100 mL·min^−1^. Bruker Esquire 3000 apparatus (Bruker Daltonik, Billerica, MA, USA) equipped with an electrospray ionization source plus an ion trap analyzer; and coupled to an Agilent 1100 capillary Liquid-Chromatography (LC) system (Agilent Technologies, Santa Clara, CA, USA) was used to perform mass spectrometry. The sample was diluted in water/methanol (1:1) solution and the analysis were carried out by FIA (flow injection analysis) with a 0.1% formic acid/methanol (1/1) eluent at a rate flow of 0.1 mL·min^−1^.

### 2.4. Magnetic Characterization of the Oleic Acid-Coated IONP (OA-IONP) and the Glycosides-Coated IONP Micelles GC22-IONP, IG20-IONP, TFA-GC22-IONP

Magnetic characterization of the samples was carried out in a vibrating sample magnetometer using 100 μL of solution in a special sample holder. Magnetization curves were recorded at room temperature by first saturating the sample in a field of 1 T. The magnetization values were normalized to the amount of iron to yield the specific magnetization (emu/g Fe). The initial susceptibility of the suspensions was measured in the field range ±100 Oe, and the saturation magnetization values (Ms) were evaluated by extrapolating to infinite field the experimental results obtained in the high field range where the magnetization linearly increases with 1/H. For determination of the Nuclear Magnetic Resonance (NMR) relaxometric values, the T2 and T1 relaxation times were measured at 37 °C in a Bruker MQ60 (Bruker Biospin, Germany) operating at 1.5 Tesla with a T2 Carr-Purcell Meiboom Gill and T1 Inversion-Recovery spin echo pulse sequences. The relaxation rate *R*_i_ (1/*T*_i_, *i* = 1, 2) values, obtained from the measured relaxation times (*T*_i_, s) were corrected by subtracting the relaxation rate of the water used to prepare the contrast agent. Linear fitting of the data and resulting slopes provide the relaxivities (r_i_, s^−1^·mM^−1^) values related to the iron concentration (mM): *R*_i_ = Rb_i_ + r_i_ [Fe].

### 2.5. In Vitro Activity of the Glycosides-Coated IONP Micelles, Inhibition of A549 and C6 Tumor Cell Proliferation

Human A549 and C6 rat glioma cell lines were maintained at 37 °C and 5% CO_2_ humidified atmosphere in Dulbecco’s modified Eagle’s medium (DMEM) (Sigma-Aldrich, St. Louis, MO, USA) supplemented with fetal bovine serum (FBS, 10%; GLinus, Madrid, Spain), glutamine (2 mM), penicillin (50 IU/mL), and streptomycin (50 mg/mL). Cells were seeded on 96-well plates (Beckton Dickinson, Le Pont de Claix, France), in DMEM complete medium, at a density of, respectively, 1.5 × 10^4^ cells/well for C6 and with 5 × 10^3^ cells/well for A549 cells. These exponentially growing cells were left to attach in some conditions and then, the medium was replaced with 100 μL of fresh DMEM without FBS and maintained overnight. Stock solutions in 50 mM ethanol of the free glycosides (GC22, IG20, TFA-GC22) or stock phosphate buffered saline (PBS) solutions of the glycosides-coated IONP micelles (GC22-IONP, IG20-IONP, TFA-GC22-IONP) were finally dispersed in DMEM complete medium for the different treatments. The cells were treated with serial dilutions (2 to 400 μM) of glycoside derivatives (final ethanol concentration < 0.8%), micelles or control vehicles (PBS) for 48 h. For the experiments with micelles, several 96-well plates were placed over a strong magnetic field provided by a Magneto FACTOR-96 (Chemicell) to increase the proximity of the nanoparticles to the cells. Similarly to previous described work [33], azelaic acid-coated IONP (Az-IONP) were tested as negative control. Cell proliferation was evaluated with a 3-(4,5-dimethyltiiazol-2-yl)-2,5-diphenyltetrazolium bromide (MTT) assay (Sigma-Aldrich). For this test, after 48 h treatment with each compound, the medium was replaced by 100 μL of fresh DMEM without phenol red containing 5 mg/mL MTT solution, and the cells incubated at 37 °C with 5% CO_2_ for additional 3 h. After this step, the medium was removed, and the resulting insoluble product of this assay precipitated as formazan was dissolved in 100 μL of DMSO, and the optical density of the solution was measured in a Spectramax Plus equipment (Molecular Devices Corporation, San José, CA, USA) at 595 nm. Three independent experiments were performed with each compound tested in triplicates. The formula used to calculate the percentage of proliferation inhibition was:

% inhibition = 100 − 100[(*X* − B)/(A − B)]

where A is the formazan produced by cells maintained in DMEM complete medium (high mitosis control), B is the produced by cells in DMEM without FBS (low mitosis control), and *X* represents the optical density of cells treated with test inhibitors. Dose response plots of percent inhibition versus concentration were adjusted to sigmoidal curves from which IC_50_ values were calculated using Graph Pad Prism 5.0 software. Using this formula, 100% inhibition means that the treated cells produced the same formazan as the cells in DMEM without FBS (low mitosis control). If the percentage is higher than 100% means that the cells are dying.

## 3. Results

### 3.1. Synthesis of Glycosides-Coated IONP Micelles

**Figure 1:**
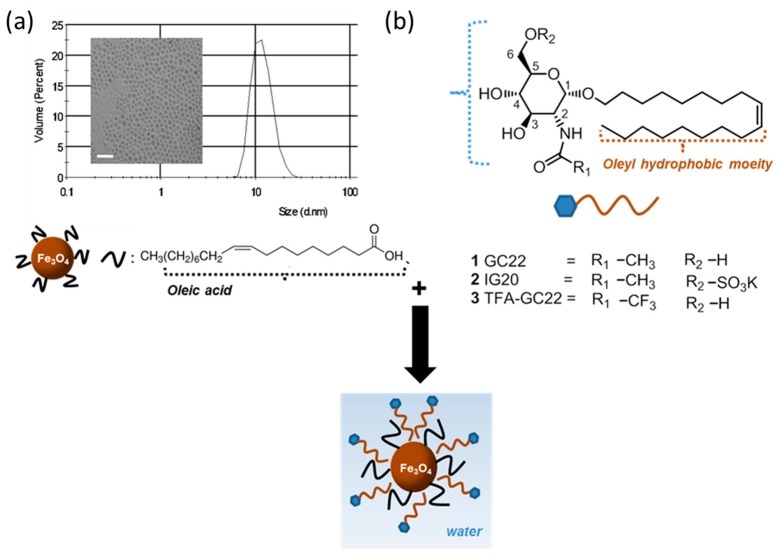
Scheme of the synthesis of the glycosides-coated IONP micelles. (**a**) Hydrodynamic size and TEM image (scale bar: 25 nm) of OA-IONP; (**b**) Structure of the bioactive glycosides.

OA-IONP were synthetized at high temperature by thermal degradation of iron organic precursors blended with the OA surfactant. The resulting OA-IONP colloidal solution in hexane was stable and monodisperse, with a 0.25 polydispersity index and a 10 ± 3 nm mean hydrodynamic size; also, iron oxide cores were spherical with a diameter of 7 ± 2 nm determined by TEM (Figure 1a). These values are comparable to previously reported IONP prepared with similar synthesis and confirmed the high control on size and uniformity of the particles provided by thermal degradation method [34,35]. The crystalline structure of the iron oxide cores was determined by X-ray diffraction (XRD) and the pattern corresponded to a magnetite phase (Powder Diffraction File Card No. 16-0629; Supplementary Figure S1a). Surfactant coating was further characterized by Fourier Transformed Infrared spectroscopy (FTIR). The spectrum displayed the vibration peaks characteristic of both, the magnetite core and the OA surfactant (Figure S1b) [36]. For the magnetite core, the band typical of the Fe–O bound in crystalline structure was seen at 590 cm^−1^. For OA, (νa C–H) and (νs C–H) corresponding to the aliphatic moieties appeared respectively at 2920 cm^−1^ and 2850 cm^−1^, and the vibrations corresponding to the carboxylic group (ν C–O) were found at 1625 cm^−1^ and 1530 cm^−1^. These last peaks also give an indication about the kind of coordination of the surfactant with the iron oxide cores. Indeed, wavelengths were higher than the typical C–O band but lower than the C=O one, and additionally the differences represented less than 110 cm^−1^. This suggest that the carboxyl is coordinated via a bidentate complex between the two oxygen and the iron atoms at the core surface. Thermogravimetric analysis (TGA) was also performed to evaluate the graft density of the organic coating. As shown in Figure S1c, the weight loss between 190 and 300 °C corresponded the OA removal. This accounted for 15% of the total weight of the nanoparticles.

Finally, one of the most important properties of the iron oxide nanoparticles that bio-applications, especially MRI, take advantage of is their magnetism. It was measured using a vibrating sample magnetometer (VSM) and OA-IONP displayed a superparamagnetism behavior with a high saturation magnetization value Ms of 70 emu·g^−1^ (Supplementary Figure S1d). This is a typical feature of the IONP prepared by thermal degradation methods that produce highly crystalline cores, compared to the aqueous co-precipitation method that produce IONP of lower saturation magnetization. This difference is particularly appreciable in the case of magnetic guidance bio-applications, where high Ms values strongly support the efficiency of magnetic attraction [15,37].

Fernández-Mayoralas et al. described a family of synthetic glycoside derivatives as inhibitors of glioma and adenocarcinoma growth, of which we selected three compounds for the preparation of OA-IONP encapsulated anti-tumor micelles (Figure 1b) [16]. In all cases, the bioactive glycosides have an oleyl chain moiety with α anomeric configuration of the glucosamine scaffold, described as optimal for antitumoral activity [24]. The antimitotic activities of GC22, IG20, TFA-GC22 against C6 (rat glioma line) and A549 (human lung adenocarcinoma line) are described in Table 1. GC22-taken as reference-presented an IC_50_ in the micromolar range for both cell lines. The influence of a different amide group (R_1_) at position C-2 (trifluoroacetamide, TFA-GC22) and the change caused by attachment of a negative hydrophilic oxosulfonyl group at C-6 position (R_2_) in IG20 were studied. Although the trifluoroacetamide group did not seem to affect much the antimitotic activity, the presence of the oxosulfonyl group had a deleterious effect on the bioactivity, with IC_50_ ten times higher, >100 μM. A more detailed information of the effects of the substituted groups on the glucosamine backbone on the inhibition of cancerous cell lines can be found in previous reports [17]. The chemistry for the preparations of the derivatives is well-known in the field and synthetic procedures can be obtained in previous publications [16,17,31].

**Figure 2:**
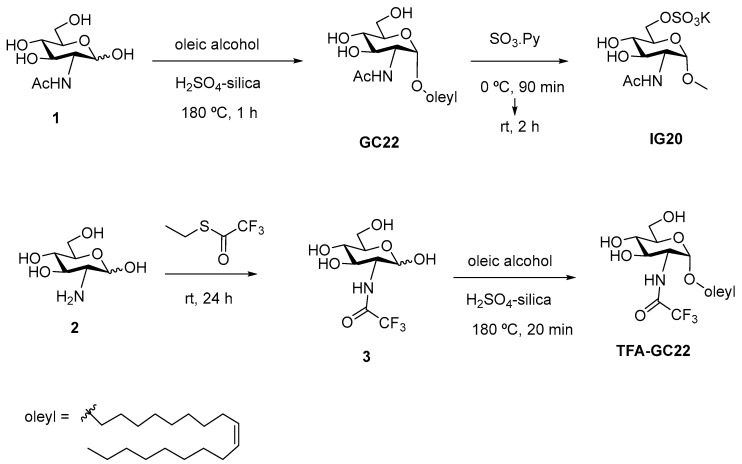
Synthesis of the glycosides GC22, IG20, TFA-GC22.

In brief, GC22 was obtained by Fischer-type glycosidation of *N*-acetyl-d-glucosamine (1) with oleyl alcohol. IG20 was prepared in a single step by regioselective sulfation of GC22 using SO_3_-pyridine complex in 81% yield. For the synthesis of TFA-GC22, d-glucosamine (2) was subjected to *N*-trifluoroacetylation followed by glycosylation with oleyl alcohol via H_2_SO_4_-silica catalyzation (Figure 2).

The hydrophobic OA-IONP were then encapsulated following a nanoemulsion method already reported [38], as micelles made of the oleyl glycosides to obtain GC22-IONP, IG20-IONP and TFA-GC22-IONP, respectively. In this process, the oleyl moiety of the glycoside intercalates with the OA aliphatic chain of the magnetite core through hydrophobic van der Waals interactions while the hydrophilic sugar counterpart covering the outer side provides the required water stability as illustrated in Figure 1 [38]. The resulting nanostructure was defined as co-dependent because such as the OA-IONP, the glycosides-except IG20-were not soluble in water and only when pairing them led to the final hydrophilic micellar suspensions. OA-IONP, in this sense, simultaneously acted as scaffold and as micelle promoter since the free glycosides did not form spontaneous micelles.

### 3.2. Physicochemical Characterization of the Glycosides-Coated IONP Micelles

**Figure 3:**
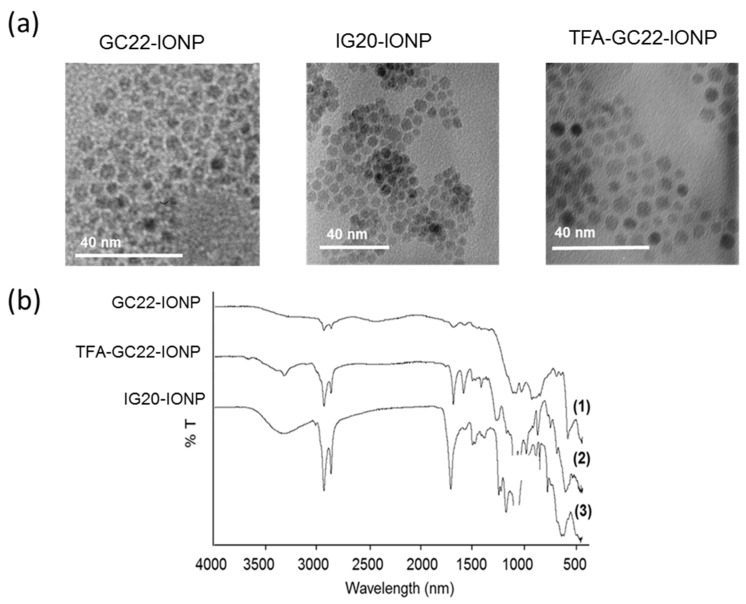
Characterization of the glycosides-coated IONP. (**a**) TEM images and (**b**) FTIR spectra of the three glycoside-coated IONP.

The hydrodynamic sizes of the micelles were in the 50 nm range of (Table 2) with pdi below 0.25, the standard level to be considered as a monodisperse population. TEM results confirmed that the NPs were well-dispersed with no aggregation (Figure 3a). However, the increase in hydrodynamic micellar sizes probably indicates the encapsulation of a few OA-IONP within the same micelle or a slight aggregation in solution of different micelles. The absolute values of zeta potential were in all cases above 25 mV at 7.4 pH (Table 2), indicating a good stability of the micellar colloidal dispersions by electrostatic repulsion. GC22-IONP micelles show a ζ potential around −25mV attributable to the glucosamine layer. IG20-IONP micelles had a higher negative −50 mV ζ potential because of the additional presence of the negatively charged oxosulfonyl group on the glucosaminic scaffold. Surprisingly TFA-GC22-IONP micelles had a positive ζ which might be explained by the de-acetylation and formation of a positive amine group at C2 during the nanoemulsion process. Fourier Transformed Infrared (FTIR) spectra of the micelles (Figure 3b) confirmed the presence of the sugars on the outer layer. The vibration of the oleyl glucosamides were observed at 620, 2920 and 1700 cm^−1^, in addition to the peaks characteristic of OA-IONP (for instance the stretching band at 590 cm^−1^ for the Fe–O bound of the inorganic crystalline core structure). Partial formation of the amine derivative of TFA-GC22 in the coating of the TFA-GC22-IONP micelle was also verified, FTIR spectra showing a strong absorption band at 3400 cm^−1^, corresponding to the primary amine vibration (ν C–NH_2_). This was also confirmed by the molecular weight of the adduct of the glucosamine de-acetylated detected by mass spectroscopy (MS) (Supplementary Figure S2).

The magnetic and relaxation properties of IONP micelles are important features for their applications as magnetically delivered drug platforms and as MRI contrast agents. The NMR relaxometric properties at 1.5 T and 37 °C were then investigated, and the longitudinal and transverse relaxivities (*r*_1_, *r*_2_, respectively) calculated in water suspensions. The results were similar for each micelle with high *r*_2_ (from 140 to 200 s^−1^·mM^−1^) and small *r*_1_ (below 4.5 s^−1^·mM^−1^); values particularly appropriate for T_2_-weighted MRI-based contrast agents (Table 1) [39].

### 3.3. In Vitro Antitumoral Activities of the Glycosides-Coated IONP Micelles

We assessed whether the glycosides under IONP-based micellar formulation have preserved their antimitotic activity for C6 rat glioma and A549 human lung carcinoma cells in culture. GC22-IONP, IG20-IONP and TFA-GC22-IONP were synthesized with a final amount from 2.5 to 3.5 mg·mL^−1^ of these molecules in the micellar formulation. To calculate the concentration of the glycosides in the colloidal dispersion, lyophilized micelles were partitioned by sonication in a water/ethanol mixture. After centrifugation, supernatants containing the free glycosides were quantified by high performance liquid chromatography (Table 2).

The cells were treated with dilutions of the micelle solution and cell growth was evaluated after 48 h incubation by MTT assays. Control NP consisted of OA-IONP that underwent an oxidative treatment to get azelaic acid-coated IONP (Az-IONP), stable in water [40]. The inhibitory activities of the micelles and the control on rat glioma and lung carcinoma cells with respect to the molar concentration of the free glycosides are shown respectively in Supplementary Figure S3 and S4. These inhibitory activities were also determined in the presence of a magnetic field placed outside the bottom of each well in the plate. The results for the 50% inhibitory dose values (IC_50_) compared to the IC_50_ for the free glycosides were summarized in Table 1.

All the glycoside-coated IONP micelles inhibited proliferation of the both cancer cell lines with IC_50_ in the micromolar range while control Az-IONP did not displayed any inhibition activity. In general, the inhibitory concentration for all micellar preparations was within the same range but slightly higher as the corresponding free glycoside used in the formulation. As an exception to this pattern, IG20-IONP had higher inhibitory activity in micellar form than as a free glycoside. In fact, the free IG20 glycoside did not have any inhibitory activity in C6 cell line at the highest concentration tested (100 μM). Among the micellar preparation, the greatest antimitotic activity was obtained for TFA-GC22-IONP (with a fraction of amine derivatives in the coating) on rat glioma cells. As control, the inhibitory activity of the free amine derivative of TFA-GC22 was also assessed and gave similar results than its parent compound. It can also be seen that when the biological activities were determined in the presence of a magnetic field, the results were similar or worse. This may be explained because the magnet immediately attracts the NP to the center of the plate so they cannot disperse over the whole plate.

## 4. Discussion

We have synthesized a variety of theranostic micelles formed by OA-IONP stabilized in water by a coating made of different antitumoral oleyl glycosides. These original nanoparticles were originally prepared at high temperature by thermal degradation of organic iron precursors resulting in iron oxide cores with higher quality to those obtained by hydrolytic routes as suggested by previous studies [9,41], and ready to be used as contrast agent and magnetically driven carrier. Three oleyl derivative glycosides previously reported as antitumoral agents [16,17] were then used to create amphiphilic structures where the oleic acid surfactant of the NP and oleyl moiety of the sugars associated as a Van der Walls interaction pair, while the hydrophilic part of the glycoside stood in the outer layer for aqueous stabilization. We obtained this micellar-like structure by spontaneous arrangement of the two moieties using a nanoemulsion procedure [38]. This approach is currently used for synthesis of other NP-based amphiphilic assemblies as it speeds up and simplified this preparation and provides reproducible and highly uniformed dispersion especially when compared to the reverse evaporation method highly extended in the field [42].

Our drug delivery approach is different from normal controlled drug delivery systems (e.g., liposomal or polymeric NP), in which the drug is usually incorporated by encapsulation in a carrier vehicle or by ionic or covalent bindings onto a prior hydrophilic NP coating [27]. This formulation facilitates the diffusion and releasing of the drug by passive transport or enzymatic cleavage once the NP has reached to the site of therapeutic interest. Additional active functionalization with peptides, proteins or antibodies lead to enhanced targeting multifunctional formulations for a very efficient delivery [11]. Here, the addition of an active targeting ligand on these peculiar micelles is a synthetic challenge. The main aspect of our solution lies in the fact that the stabilizing and biocompatible coating of the NP is the own active component of the drug, and therefore should maintain its therapeutic efficiency. For this reason, we verified that the antitumoral activity was fully preserved upon the micelle formation. The presence of an oleyl aglycon group on the glycosides was described as the most positive factor for cancerous cell growth inhibition. Based on our previous work, the effect of these glycosides seems to be the result of their interaction with the cell membranes. It was previously shown that GC22 and TFA-GC22 act altering the sphingolipid metabolism of cancer cells [20] by activating neutral sphingomyelinase at the cell membrane [17]. In another work, we showed that the fluorescent derivative of IG20 was predominantly located in the membrane of chromaffin cells [43]. In these OA-IONP-based glycosidic micelles, the aliphatic chain is shielded by the amphiphilic nanoassembly. Of notice, although this could also proceed against our own interest, bioactivity was preserved and in the IG20-IONP case was even higher than the one of the free molecules. This may indicate that even under a micellar form, the bioactive glycosides can intercalate in the lipid bilayer of the cell membrane. If for the free glycosides, a higher hydrophilicity is inclined to reduce the antimitotic activity for the both, C6 and A549 cancer cell lines (IC_50_ values were much higher for the only water-soluble glycoside IG20, while GC-22 and TFA-GC22 presented good and almost similar inhibitory activities), the opposite effect was observed when glycosides are under micellar form. Indeed, inhibition of cell proliferation by IG20-IONP got a significant increase and the activities of TFA-GC-22-IONP (considering the partial formation of the hydrophilic amine derivative) were relatively better preserved than the GC22-IONP ones. Therefore, it could be the case that the micellar state of IG20 in the NP could enable the intercalation of IG20 in the membrane, similarly to some amphiphilic surface active drugs, thus facilitating its biological activity [44]. Besides, the free oleyl glycosides were successfully used in vivo bound to the BSA protein, a fatty acid transporter which plays a role similar to that played by the micelles described here [24].

One of the main challenges of anti-cancer chemotherapy is that it might affect both tumor and healthy tissues. The optimal solution will be synthesizing drugs specifically addressed against cancerous cells taking advantage of the differences between the tumor and normal cell biological pathways [45]. New specific targeting strategies including drug delivery platforms (either biological such as targeting organic ligands, or physical such as magnetically guided NP) start to provide different alternatives. In the present work, oleyl glycosides act on the activated lipid synthesis pathways of tumor cells, that require cell membrane products to sustain transformed cell proliferation [19]. This first selective characteristic, combined with other properties of the IONP-based drug delivery platform, such as a possible EPR effect enhancing the accumulation of NP in tumors, magnetic-guided delivery, or hyperthermia applications, can be a strong support point for an in vivo chemotherapy with high specificity for the modified cells. On this regard, EPR effects shall be facilitated by the 50 nm size of the micelles, particularly adapted for an easy accumulation or diffusion through increased vascular permeability often accompanying pathological processes [46] and also by the long enough circulation times in the same order those described for IONP of similar hydrodynamic sizes [47]. This contributes to increase the chances of higher micellar accumulation and to provide a local optimal inhibitory concentration for the glycosides. In the same way, a previous evaluation of the free glycosides in vivo showed that contributors to a low efficiency of the treatment were both, the low aqueous solubility of the compounds together with their degradation by activated macrophage enzymes (i.e., hexosaminidases) present in the inflamed tumor area, that can cleave the glycoside bonds and inactivate the inhibitor [24]. Therefore, it would be of great interest to study whether glycosides under the micellar form could cultivate an enzymatic resistance toward hexosaminidases degradation, contributing as well to higher efficiency of the antitumoral activity. Lastly, it is important to point out the concern about potential toxicity of iron oxide nanoparticles-based therapeutics still under debate, and that may limit their use in vivo. Although many of the first developed IONP contrast agents have been withdrawn from the market because of their low benefice-to-risk balance, a new generation of iron oxide formulations is on the market or under phase III clinical trials either for medical imaging or therapy, particularly fueled by hyperthermia and lymph node detections [48,49]. The perspective of translational research for the next decade is quite optimistic if lessons from the previous uses are learned and toxicity assessments are performed on case-by-case basis [50,51]. Regarding the specific IONP-based micellar structures, toxicity data are still limited and only available from pre-clinical studies, suggesting similar behavior for glycoside-coated IONP micelles [38,42,52].

## 5. Conclusions

It is widely known in the anti-cancer therapeutic field that glycosides attached to long oleyl hydrophobic chains show antimitotic activity on different cancerous cell lines. The delivery of these small molecules to the tumor site is difficult due to both their poor solubility and their susceptibility to in vivo enzymatic degradation. We have presented in this work a new nanoparticle-based micellar formulation for drug delivery in which these antimitotic molecules are used to stabilize oleic acid-coated iron oxide nanoparticles. These micelles showed high aqueous solubility, while retaining bioactivity when assessed against glioma C6 and carcinoma A549 cell lines. For theranostic applications, we showed that these micellar agents are also suitable as MRI contrast agents, grounded in the IONP magnetic properties. Taking all these results together, new opportunities for the therapeutic use of anti-tumor oleyl glycoside are open with extended in vivo applications, such as MRI-based diagnoses or magnetic-guided delivery. Further in vivo studies are needed to validate the pharmacological potential of these micellar candidates, in particular pharmacokinetics experiments. Finally, our study also contribute to the widespread development of nano-formulations in which the stabilizing coating and therapeutic function should go hand in hand.

## 6. Patents

Patent EP 2921179 A1 is resulting from the work reported in this manuscript.

## Figures and Tables

**Table 1:** Comparison of the IC_50_ of the free glycosides and the glycosides-coated IONP micelles. w/o: without magnet; w/: with magnet.

Compound	IC_50_ (μM) C6	IC_50_ (μM) A549
*GC22*	15.5 ± 0.3	10
*GC22-IONP w/o magnet*	55.0	100.5
*GC22-IONP w/ magnet*	70.0	95.0
*IG20*	>100	97
*IG20-IONP w/o magnet*	68.5	64.4
*IG20-IONP w/ magnet*	57.2	91.0
*TFA-GC22*	14.2 ± 0.3	8.6
*TFA-GC22-IONP w/o magnet*	24.4	40.3
*TFA-GC22-IONP w/ magnet*	49.8	42.0

**Table 2:** Main physicochemical characteristics of the glycosidic IONP micelles.

Glycosidic IONP Micelles	Size (nm)	Pdi	Zeta Potential (mV)	[Fe] (mg/mL)	C° [glyco] (mg/mL)	Relaxometric Parameters (s^−1^·mM^−1^)
GC22-IONP	40.5	0.24	−27	0.6	2.5	*r*_1_ 2.7 *r*_2_ 140
IG20-IONP	52.2	0.15	−42	1.1	3.1	*r*_1_ 4.4 *r*_2_ 195
TFA-GC22-IONP	49.1	0.17	+53	0.3	2.5	*r*_1_ 3.6 *r*_2_ 137

## Supplementary Materials

The following are available online at http://www.mdpi.com/2079-4991/8/8/567/s1, Figure S1: Characterization of the oleic acid-coated IONP (OA-IONP). Figure S2: Mass spectra of TFA-GC22-IONP performed in positive ionization. Figure S3: Inhibitory activities of the free glycosides and the glycosides-coated IONP micelles with respect to their inhibitory concentration (molar concentration) for rat glioma cells (C6) after 48 h incubation with or without (w/o) the support of a magnetic field placed at the bottom of the plate. Figure S4: Inhibitory activities of the free glycosides and the glycoside-coated IONP micelles with respect to their inhibitory concentration (molar concentration) for lung carcinoma cells (A549) after 48 h incubation with or without (w/o) the support of a magnetic field placed at the bottom of the plate.
