# Supplementary material for: Micellar Iron Oxide Nanoparticles Coated with Anti-Tumor Glycosides
Source: Nanomaterials (Basel). 2018 Jul 25;8(8):567. doi: 10.3390/nano8080567 (PMC6116232; doi:10.3390/nano8080567)
Supplement: Supplementary file 1 [file nanomaterials-08-00567-s001.zip › nanomaterials-320190-SI.pdf]

Supplementary Materials

Micellar iron oxide nanoparticles coated with antitumor glycosides

Hugo Groult [1], Isabel García-Álvarez [2,3], Lorenzo Romero-Ramírez [4], Manuel Nieto-Sampedro [4,5], Fernando Herranz [6], Alfonso Fernández-Mayoralas [7] and Jesús Ruiz-Cabello [8,9,*]

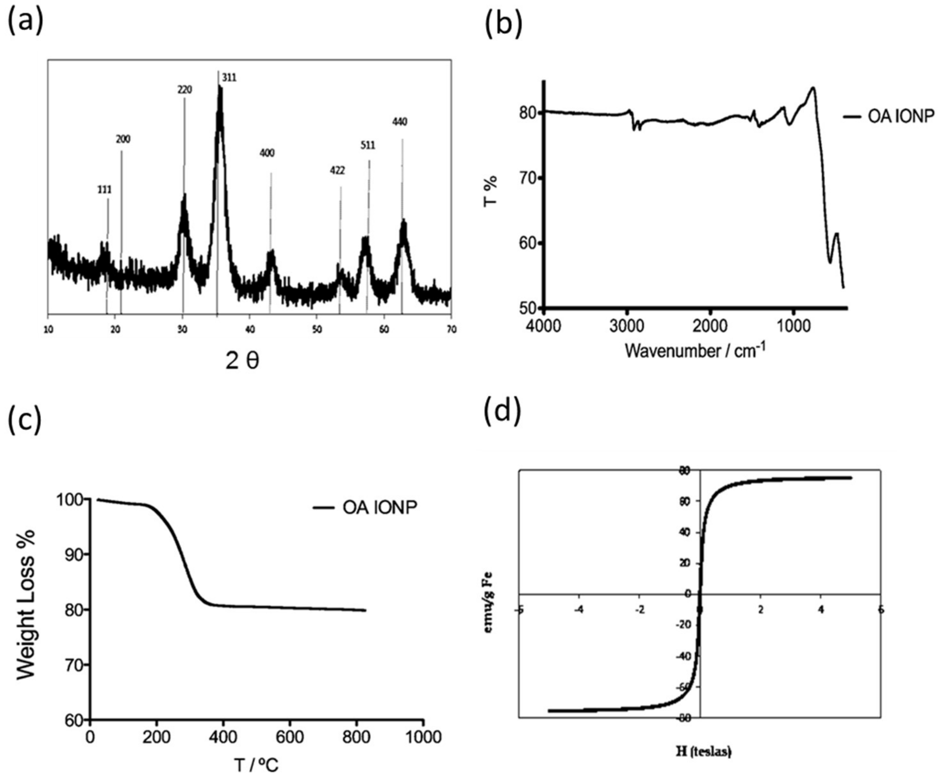

Figure S1. Characterisation of the oleic acid coated IONP (OA-IONP). (**a**) XRD spectra; (**b**) FITR spectra; (**c**) thermogravimetric analysis (TGA) and (**d**) Magnetisation curve at 298 K (1 Oe = 10 000 T) of OA-IONP.

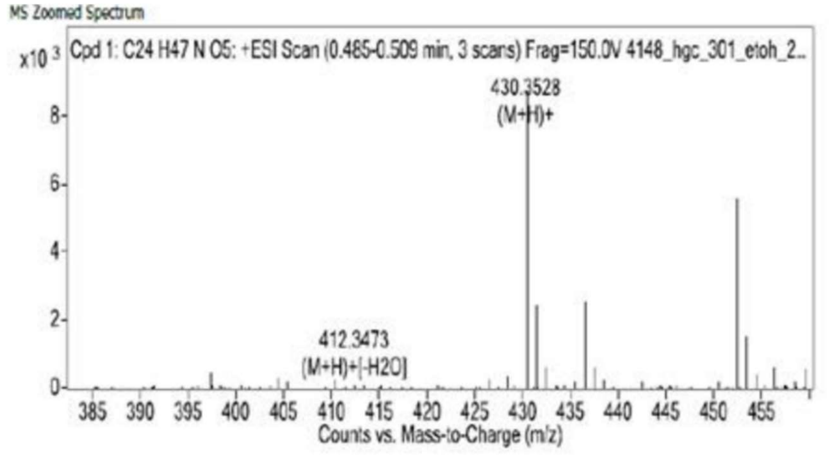

Figure S2. Mass spectra of TFA-GC22-IONP performed in positive ionization.

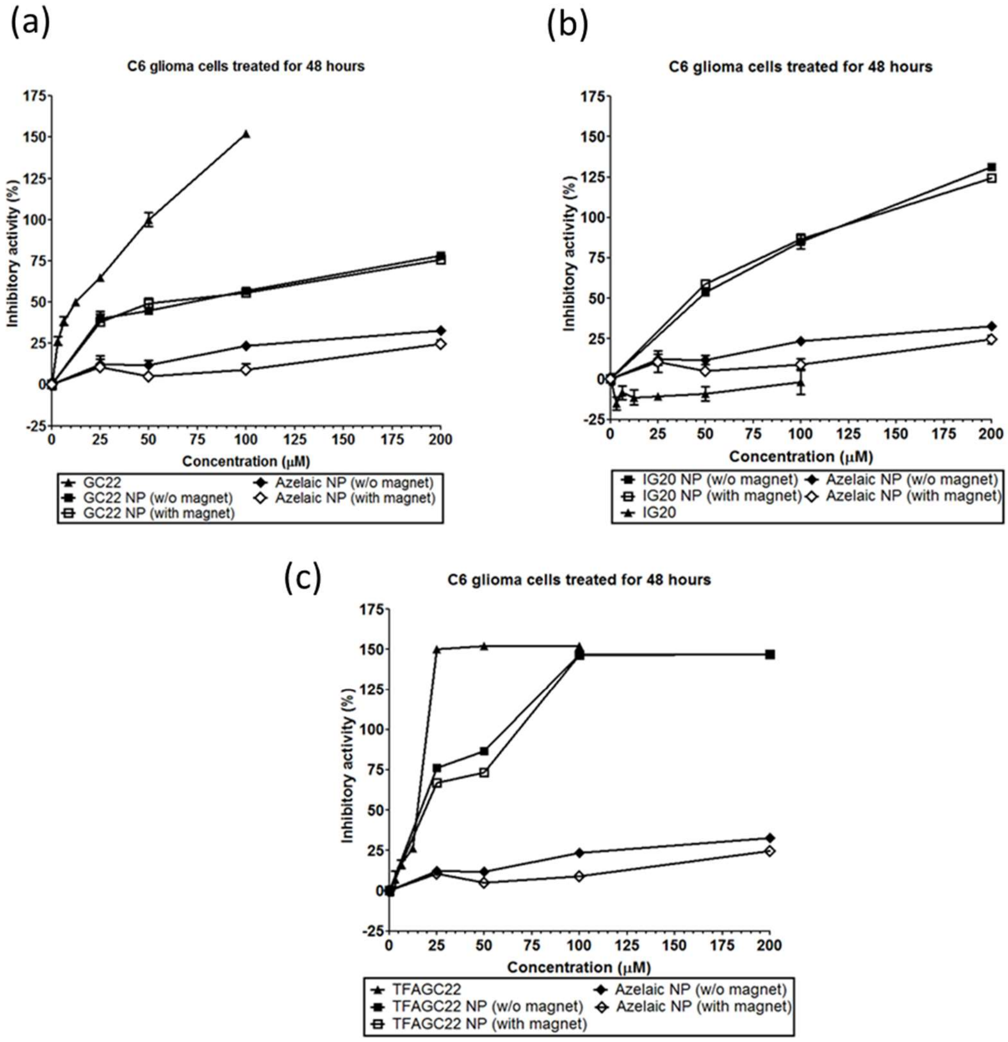

Figure S3. Inhibitory activities of the free glycosides and the glycosides coated IONP micelles with respect to their inhibitory concentration (molar concentration) for rat glioma cells (C6) after 48 hours incubation with or without (w/o) the support of a magnetic field placed at the bottom of the plate. (**a**) GC22 and GC22-IONP; (**b**) IG20 and IG20-IONP; (**c**) TFA-GC22 and TFA-GC22-IONP. The results are represented as the mean ± SEM of three independent experiments, each in triplicates for the free glycosides and the mean of one experiment in triplicate for the glycoside-coated IONP micelles.

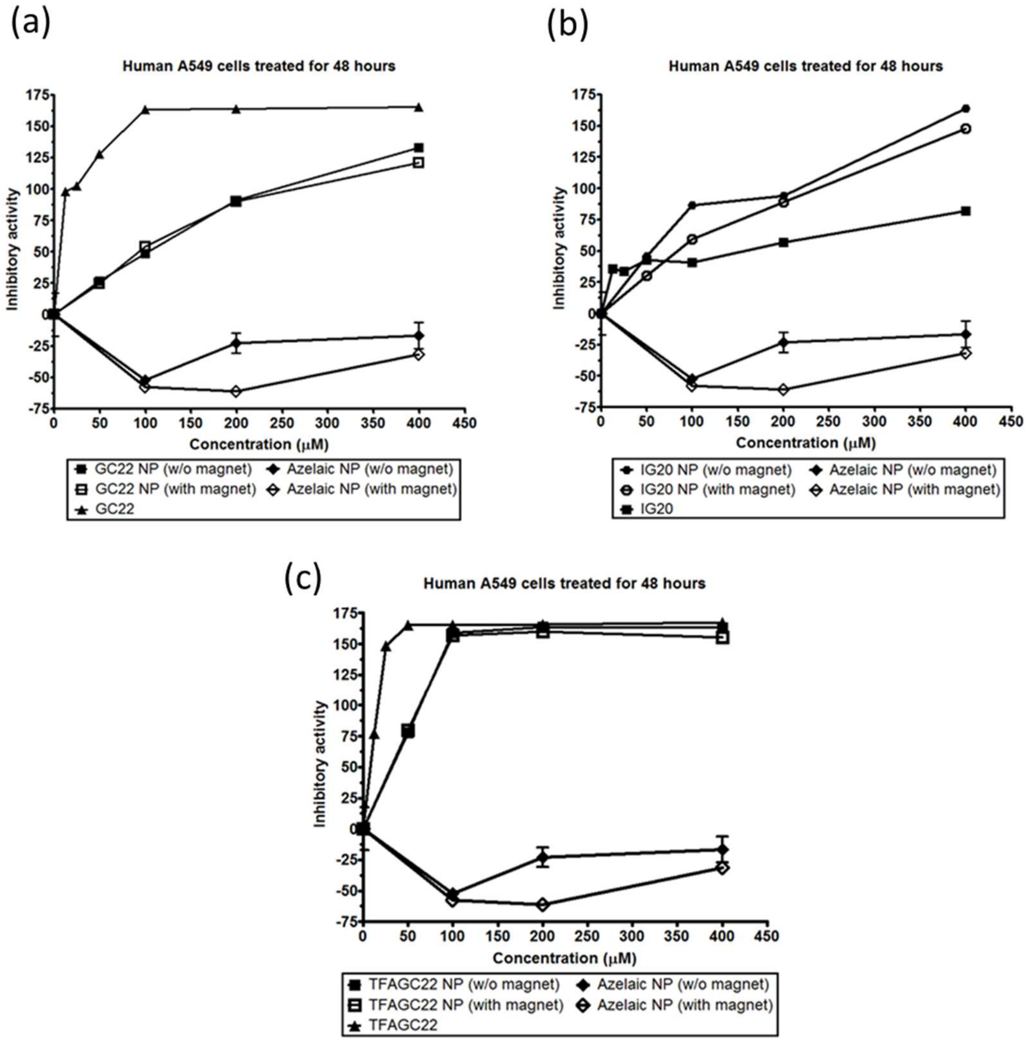

Figure S4. Inhibitory activities of the free glycosides and the glycoside-coated IONP micelles with respect to their inhibitory concentration (molar concentration) for lung carcinoma cells (A549) after 48 hours incubation with or without (w/o) the support of a magnetic field placed at the bottom of the plate. (**a**) GC22 and GC22-IONP; (**b**) IG20 and IG20-IONP; (**c**) TFA-GC22 and TFA-GC22-IONP. The results are represented as the mean ± SEM of three independent experiments, each in triplicates for the free glycosides and the mean of one experiment in triplicate for the glycoside-coated IONP micelles.
